# Earlier continuous renal replacement therapy is associated with reduced mortality in rhabdomyolysis patients

**DOI:** 10.1080/0886022X.2022.2132170

**Published:** 2022-10-19

**Authors:** Xiayin Li, Ming Bai, Yan Yu, Feng Ma, Lijuan Zhao, Yajuan Li, Hao Wu, Lei Zhou, Shiren Sun

**Affiliations:** a Department of Postgraduate Student, Xi’an Medical University, Xi'an, China; b The Nephrology Department of Xijing Hospital, The Fourth Military Medical University, Xi’an, China; c The Clinical Laboratory Department of Xijing Hospital, The Fourth Military Medical University, Xi’an, China

**Keywords:** Rhabdomyolysis, acute kidney injury, continue renal replacement therapy, mortality, creatine kinase

## Abstract

**Background:**

Continuous renal replacement therapy (CRRT) is commonly employed for rhabdomyolysis (RM) patients. However, the optimal initiation timing of CRRT and prognostic factors were not well evaluated for patients with RM. We aimed to investigate the efficacy of CRRT timing on mortality and the risk factors for death in RM patients who received CRRT.

**Methods:**

RM patients who received CRRT between 1 May 2010 and 31 May 2021 in our center were retrospectively included. Univariate and multivariate logistic analyses were performed to identify the risk factors for primary outcome (90-day mortality).

**Results:**

A total of 134 patients were included in our study. The 90-day mortality rate was 38.06%. The median time from CRRT initiation to peak CK occurrence was 4.8 h (IQR −16, 14), 67 patients received CRRT before 4.8 h after peak CK occurrence (early CRRT), and 67 patients received CRRT beyond 4.8 h after peak CK occurrence (late CRRT). Multivariate logistic regression analysis showed that the time from CRRT initiation to the peak CK (per 1 h, OR 1.026, 95% CI 1.004–1.049, *p* = 0.023), late CRRT (OR 3.082, 95% CI 1.072–8.859, *p* = 0.037), elevated serum cardiac troponin I (cTnI) (per 1 ng/mL, OR 1.218, 95% CI 1.011–1.468, *p* = 0.038), older age (per 1 year, OR 1.042, 95% CI 1.003–1.081, *p* = 0.032), and need of mechanical ventilation support (OR 4.632, 95% CI 1.292–16.61, *p* = 0.019) were independent risk factors for 90-day mortality.

**Conclusions:**

Earlier CRRT initiation before 4.8 h after peak CK occurrence was associated with lower 90-day patient mortality.

## Background

Rhabdomyolysis (RM) is a complex clinical syndrome characterized by damage to skeletal muscle cells and the release of cellular contents such as myoglobin (MB), creatine kinase (CK), and electrolytes into the blood circulation. A variety of medical and surgical conditions can lead to the development of RM and it is particularly common in patients with trauma and heat stroke, with the morbidity reported as high as 31% [1] and 34% [2,3], respectively. In addition to muscle damage, RM can induce a series of life-threatening complications. Acute kidney injury (AKI) is the most common and severe complication of RM, and 20–60% of patients experience AKI. For RM-related AKI patients, the mortality rate is significantly increased and was reported to be as high as 60% [4,5].

The elevated circulating MB, which could accelerate kidney injury by promoting vasoconstriction, blocking renal tubules, causing oxidative stress lipid peroxidation, and inducing inflammation, is considered to be the most important pathogenesis of RM-related AKI [6–8]. Traditional treatments for the prevention of RM-related AKI include the blockade of causative factors and early rehydration to dilute serum and urine MB concentrations [6], in addition, some emerging anti-oxidant and anti-inflammatory drugs have also been proven to protect the kidneys [9]. However, in critical RM patients who develop severe AKI with electrolyte disorder and volume overload, there are no other specific therapies available except the renal replacement therapy (RRT) [10].

It should be considered that the molecular weight of MB is 17.6 kDa, and the techniques used for classical dialysis have shown limited ability to remove circulating MB [11]. Continuous renal replacement therapy (CRRT) provides the best effectiveness for continuous removal of MB compared to peritoneal dialysis and intermittent hemodialysis modalities [10]. It has been reported that CRRT is used in 8–41% of RM patients [5,12], and it is more often used specifically in patients with hemodynamic disturbances [13]. Convection is better at removing solutes with larger molecular than diffusion [10], and previous studies have reported successful clearance of circulating MB by the continuous veno-venous hemofiltration (CVVH) modality [14,15]. In an animal study, early CVVH was proven to effectively clean MB, improve kidney mitochondrial function and inhibit apoptosis [16]. Moreover, it was reported that CRRT was effective in the correction of electrolyte disturbance, the removal of inflammatory mediators, and stabilization of the internal environment [14,15,17].

Although there are many theoretical benefits, the actual therapeutic effectiveness of CRRT is controversial. In a systematic review of the benefits of CRRT in patients with RM, Zeng et al. found that although creatinine, MB, and electrolyte levels improved in patients with CRRT, mortality remained unchanged [18]. Due to the absence of compelling evidence that the early application of CRRT improves the prognosis of RM patients and taking into account the potential risks of CRRT, in clinical practice, it is crucial to consider all confounding aspects of the patient’s disease and individualize treatment [19]. Researchers do not recommend the routine use of CRRT in RM patients to prevent AKI [20]. In the recommendation from current management guidelines of RM, CRRT should be initiated in patients who developed severe AKI complicated by fluid or electrolyte disturbances not responsive to pharmacological therapy [21]. However, for crush injury and heat stroke patients, two common causes of RM, researchers have suggested that CRRT should be more aggressively adopted due to the aforementioned advantages [22,23]. These controversial opinions on the time of CRRT initiation are confusing for clinicians.

To the best of our knowledge, no study has directly investigated the timing of CRRT initiation in RM patients. Therefore, we conducted a retrospective study on RM patients who received CRRT, to evaluate the timing of CRRT initiation on patient mortality and analyze the risk factors for patient mortality.

## Methods

### Population and setting

This retrospective cohort study was performed in the intensive care unit (ICU) and medical ward of Xijing Hospital from 1 May 2010 to 31 May 2021. Patients who were diagnosed with RM (CK >1000 IU/L) with a typical clinical presentation or etiology predisposing them to RM, and received CRRT during hospitalization were included. Patients with any of the following criteria were excluded: (1) age <18 years; (2) chronic kidney disease (CKD) diagnosed before admission; (3) elevated CK caused by acute myocardial infarction (AMI); (4) CRRT initiation before hospital admission to our center; (5) missed patients. The ethics committee of Xijing Hospital was informed and approved this retrospective study (approval number: KY20213020-1). Written informed consent for participation in this study was waived due to the retrospective, noninterventional study design. All patient data were kept confidential.

### Data collection

Patient data were collected from electronic medical records. Demographic data included age, sex, comorbidities (hypertension, diabetes, cardiovascular disease), and admission diagnosis. Laboratory data included leukocytes, hemoglobin, platelets, albumin, globulin, bilirubin, prothrombin time (PT), cardiac troponin I (cTnI), calcium, potassium, and biomarkers of renal function (creatinine, eGFR) on hospital admission and before CRRT initiation. AKI was diagnosed and graded according to the 2012 KDIGO AKI guideline [24]. The severity of the disease was evaluated by the Acute Physiology and Chronic Health Evaluation II (APACHE II) score and the Sequential Organ Failure Assessment (SOFA) score. Generally, the CK value is tested once a day and adjusted according to the patient’s condition. To determine the time of peak CK, the time of each CK blood sampling during every patient’s hospitalization was recorded, if there were multiple CK peaks, the time of the first peak CK occurrence was recorded. According to the median time from CRRT initiation to the peak CK occurrence, patients were divided into an early CRRT group and a late CRRT group. The start and stop time of CRRT, treatment duration, anticoagulation modalities, and CRRT dose were recorded. The net ultrafiltration intensity (NUF) was calculated using the formula proposed by Naorungroj et al. [25]. NUF was classified as low, medium, or high intensity according to the findings of Murugan et al. [26].

### CRRT protocol and primary outcome

The professional nephrologist assessed whether CRRT should be applied based on the following criteria: patients who developed severe AKI, severe electrolyte disturbances, volume overload, acid–base disturbances, and multi-organ failure. All of the CRRT modes were CVVH or continuous veno-venous hemodiafiltration (CVVHDF) and were performed using the Gambro instrument with AN 69 dialyzer (M100, surface area 0.9 m^2^; Gambro Renal Products, Lakewood, CO). The CRRT dose was targeted on 20–25 mL/kg/h. The physician chooses the appropriate anticoagulation modes according to the KDIGO recommendations. In patients with trauma and combined high-risk bleeding, regional citrate anticoagulation was the first choice for anticoagulation. The primary outcome was 90-day mortality. Patients who survived at discharge were followed up by telephone. The long-term survival and renal outcome of the patients were assessed and recorded.

### Statistical analysis

Categorical variables were described as frequencies and percentages and tested using the Chi-square test or Fisher’s exact test. Normally distributed continuous variables were expressed as mean ± standard deviation (SD) and tested using Student’s *t*-test. Continuous variables with non-normal distribution were expressed as interquartile range (IQR) (the 25th to 75th percentile) and tested using the Mann–Whitney *U*-test. Independent risk factors of 90-day mortality were identified using logistic regression analysis. Collinearity diagnosis was employed to eliminate highly related variables. For variables with extremely skewed distributions, logarithmic transformation was performed in the analysis. The Kaplan–Meier curve was used to describe the patient accumulated survival proportion and intergroup comparisons were performed using the log-rank test. Subgroup analysis was performed according to the patient etiologies, stage of AKI, APACHE II score, and SOFA score. All statistics were calculated using IBM SPSS Statistics version 22 (IBM Corp., Armonk, NY). A two-sided *p* value <0.05 was considered statistically significant.

## Results

### Characteristics of patients with RM

**Figure 1.**
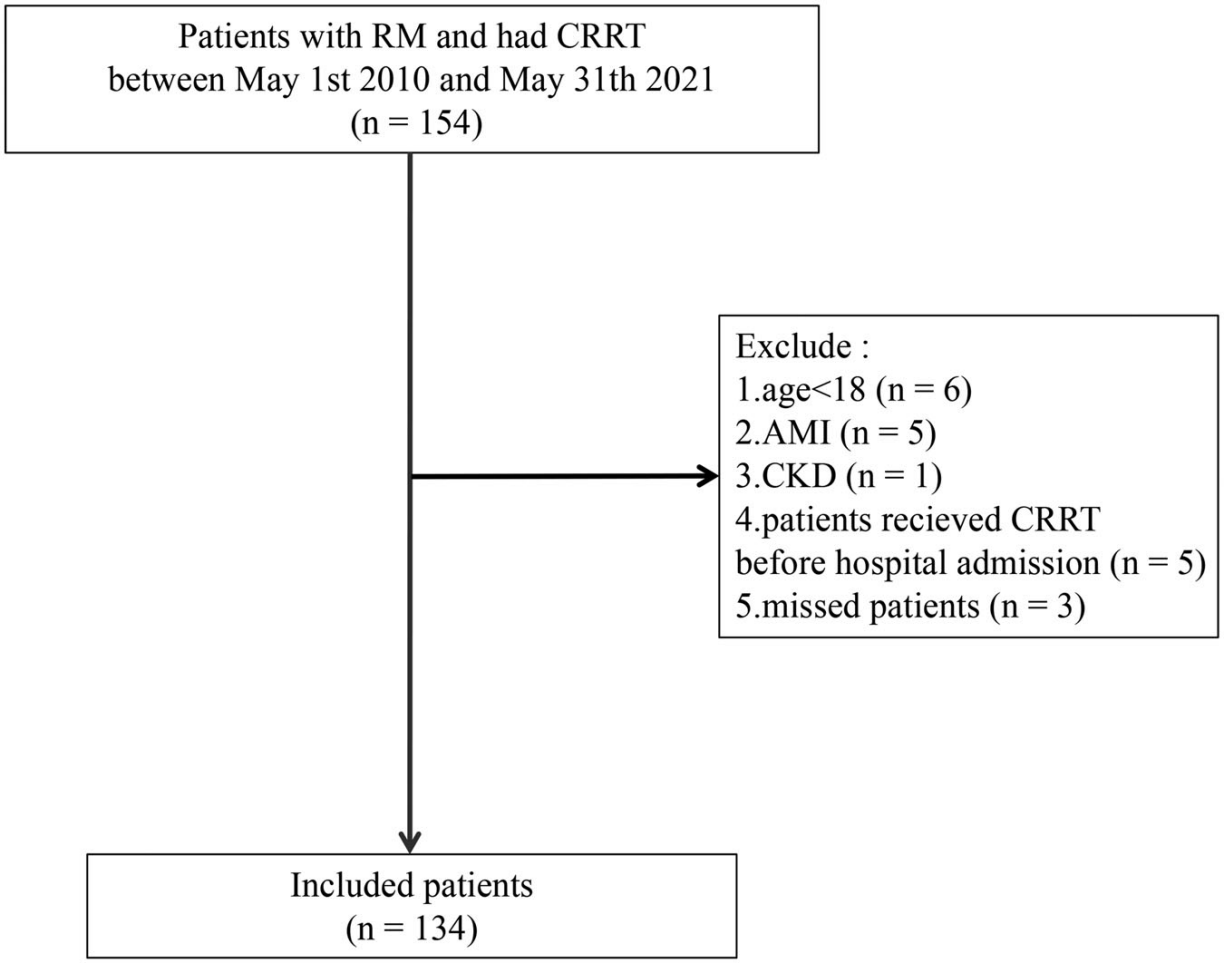
Flowchart of patient inclusion. RM: rhabdomyolysis; CRRT: continue renal replacement therapy; AMI: acute myocardial infarction; CKD: chronic kidney disease.

From 1 May 2010 to 31 May 2021, a total of 449 patients met the diagnosis of RM, including 154 (34.2%) RM patients who received CRRT, and after selection, 134 patients were enrolled in the final analysis (Figure 1).

Patient characteristics are shown in Table 1. The median age of the included patients was 49 (IQR 33.75, 62.25) years, and 112 (83.58%) patients were males. Trauma (*n* = 29, 21.64%), heat stroke (*n* = 28, 20.9%), and vascular ischemia (*n* = 19, 14.18%) were the most common causes of RM. Other causes included physical labor (*n* = 12, 9.0%), bee stings (*n* = 11, 8.21%), toxic substances (*n* = 8, 5.97%), infections (*n* = 8, 5.97%), endocrine diseases (*n* = 7, 5.22%), drugs (*n* = 6, 4.48%), surgery (*n* = 5, 3.73%), and stroke (*n* = 1, 0.75%).

**Table 1.** Characteristics of patients at hospital admission.

Variables	All patients (*n* = 134)
Age^a^	49 (33.75, 62.25)
Male (*n*, %)	112 (83.58%)
MAP (mmHg)	92.28 ± 17.75
Causes of rhabdomyolysis	
Trauma (*n*, %)	29 (21.64%)
Heat stroke (*n*, %)	28 (20.9%)
Vascular ischemia (*n*, %)	19 (14.18%)
Other (*n*, %)	58 (43.28%)
Comorbidity (*n*, %)	
Diabetes (*n*, %)	20 (14.93%)
Hypertension (*n*, %)	33 (24.63%)
Cardiovascular disease (*n*, %)	15 (11.19%)
Use of mechanical ventilation (*n*, %)^a^	61 (45.52%)
Use of vasoactive drugs (*n*, %)^a^	50 (37.31%)
Blood transfusion (*n*, %)	81 (60.45%)
APACHE II^a^	15.99 ± 8.36
SOFA^a^	7.02 ± 3.44
eGFR (mL/min/1.73 m^2^)	41.34 (21.38, 68.99)
Serum creatinine (μmol/L)	166 (103, 261.5)
Albumin (g/L)^a^	37.99 ± 8.47
Globulin (g/L)^a^	25.48 ± 4.60
cTnI (ng/mL)^a^	0.42 (0.06, 1.64)
Hemoglobin (g/L)	138.68 ± 30.41
WBC (×10^9^/L)	13.99 (9.21, 20.90)
Platelet (×10^9^/L)	147.5 (79.25, 210.75)
Calcium (mmol/L)	2.03 ± 0.29
Potassium (mmol/L)	4.12 (3.66, 4.78)
Sodium (mmol/L)	139.6 ± 10.5
Prothrombin time (s)	12.6 (11.40, 13.98)
Admission CK (IU/L)	6703.5 (1070, 25940.75)
Peak CK (IU/L)	14,293 (4974.75, 42,906)

MAP: mean arterial pressure; APACHE II: Acute Physiology and Chronic Health Evaluation II; SOFA: Sequential Organ Failure Assessment; CK: creatine kinase; cTnI: cardiac troponin I; WBC: white blood cell.

Normal range of CK: 40–200 IU/L; normal range of cTnI: 0–0.03 ng/mL; normal range of albumin: 40–55 g/L.

^a^ In univariate analysis, survivors vs. mortality, *p* < 0.05.

Of the included patients, 111 (82.84%) developed AKI before CRRT, and 44 (32.83%) developed advanced AKI (AKI II or III stage). The median eGFR before CRRT was 32.97 mL/min/1.73 m^2^ (IQR 16.89, 58.56). The medical histories included diabetes (*n* = 20, 14.93%), hypertension (*n* = 33, 24.63%), and cardiovascular disease (*n* = 15, 11.19%).

The median admission CK and peak CK were 6703.5 IU/L (IQR 1070, 25940.75) and 14,293 IU/L (IQR 4974.75, 42,906), respectively. The median time from admission to the peak CK occurrence was 29 h (IQR 16, 55), 119 (88.81%) patients reached peak CK within 72 h after admission, and the remaining patients had the peak CK within seven days after admission. The median time from admission to CRRT initiation was 28 h (IQR 21, 45.6), and 116 (86.5%) patients received CRRT within 72 h after admission. Fifty-eight (43.28%) patients received CRRT before the peak CK occurrence, and 76 (56.72%) patients received CRRT after the peak CK occurrence. The median time from CRRT initiation to the peak CK occurrence was 4.8 h (IQR −16, 14), and the patients were divided into the early CRRT group (CRRT initiation before 4.8 h after the peak CK occurrence) and the late CRRT group (CRRT initiation beyond 4.8 h after the peak CK occurrence) based the median value, with 67 patients in each group (Table 2).

**Table 2.** Variables at initiation of CRRT and outcomes of patients.

Variables at initiation of CRRT	All patients (*n* = 134)
APACHE II^a^	16.51 ± 8.16
SOFA^a^	8.38 ± 3.92
Time from admission to CRRT initiation (h)^a^	28 (21, 45.60)
Time from initiation of CRRT to peak CK occurrence^a^	4.8 (−15.6, 13.9)
Early CRRT^a^	67 (50%)
Late CRRT^a^	67 (50%)
Anti-coagulation modalities	
Regional citrate anticoagulation	68 (50.7%)
Low molecular weight heparin	37 (27.6%)
Heparin-free anticoagulation	16 (11.9%)
Others	13 (9.7%)
Net ultrafiltration intensity	
Low intensity (<1.01 mL/kg/h)	72 (53.73%)
Moderate-intensity (1.01–1.75 mL/kg/h)	26 (19.40%)
High intensity (>1.75 mL/kg/h)	36 (26.87%)
AKI stage at initiation of CRRT	
AKI 0 (*n*, %)	23 (17.16%)
AKI I (*n*, %)	67 (50%)
AKI II, III (*n*, %)	44 (32.84%)
Serum creatinine (μmol/L)	188 (115, 306.75)
eGFR (mL/min/1.73 m^2^)	32.971 (16.89, 58.56)
Prothrombin time (s)	12.8 (11.7, 14.8)
Calcium (mmol/L)	1.94 ± 0.27
Potassium (mmol/L)	4.21 (3.68, 5.04)
Sodium (mmol/L)^a^	140.1 ± 11
Hemoglobin (g/L)	134.46 ± 30.68
Platelet (×10^9^/L)	125.5 (59.75, 205.5)
WBC (×10^9^/L)	14.80 (11.08, 21.04)
Albumin (g/L)^a^	36.49 ± 7.60
Globulin (g/L)^a^	25.08 ± 4.83
cTnI (ng/mL)	0.48 (0.1, 1.99)
Outcomes	
Length of hospitalization (days)	10 (5, 18.25)
In hospital mortality (*n*, %)	20 (14.93%)
90-day mortality (*n*, %)	51 (38.06%)

CRRT: continue renal replacement therapy; APACHE II: Acute Physiology and Chronic Health Evaluation II; SOFA: Sequential Organ Failure Assessment; CK: creatine kinase; AKI: acute kidney injury; eGFR: estimated glomerular filtration rate; WBC: white blood cell; early CRRT: initiation CRRT before 4.8 h after peak CK occurrence; late CRRT: initiation CRRT beyond 4.8 h after peak CK occurrence.

Normal range of CK: 40–200 IU/L; normal range of cTnI: 0–0.03 ng/mL; normal range of albumin: 40–55 g/L.

^a^ In univariate analysis, survivors vs. mortality, *p* < 0.05.

### Univariate and multivariate analyses of patients with RM

In the univariate analysis, a higher APACHE II score, SOFA score, lower albumin, and globulin both on hospital admission and before CRRT, older age, higher cTnI on hospital admission, higher sodium before CRRT, time from admission to CRRT initiation, time from CRRT initiation to the peak CK occurrence, late CRRT, the need for mechanical ventilation support, and the use of vasoactive drugs were associated with 90-day mortality (Table 3).

**Table 3.** Univariate and multivariate logistic regression analyses of 90-day mortality of patients.

Characteristics	Univariate logistic regression		Multivariate logistic regression (model 1)		Multivariate logistic regression (model 2)	
	OR (95% CI)	*p* Value	OR (95% CI)	*p* Value	OR (95% CI)	*p* Value
Age^a^	1.04 (1.02–1.07)	0.000	1.028 (0.995–1.063)	0.101	1.042 (1.003–1.081)	0.032
Use of mechanical ventilation (*n*, %)^a^	7.63 (3.45–16.84)	0.000	4.632 (1.292–16.61)	0.019	4.861 (1.332–17.742)	0.017
Use of vasoactive drugs (*n*, %)	5.22 (2.44–11.17)	0.000	1.973 (0.542–7.187)	0.303	2.331 (0.643–8.455)	0.198
*Variables at admission to hospital*						
APACHE II	1.05 (1.00–1.09)	0.034	0.95 (0.857–1.053)	0.328	0.926 (0.827–1.036)	0.18
SOFA	1.20 (1.08–1.35)	0.001	0.913 (0.655–1.272)	0.592	0.898 (0.64–1.261)	0.536
Albumin (g/L)	0.94 (0.9–0.99)	0.009	0.946 (0.815–1.098)	0.464	0.963 (0.828–1.119)	0.619
Globulin (g/L)	1.12 (1.03–1.22)	0.007	1.156 (0.909–1.47)	0.238	1.175 (0.922–1.498)	0.192
cTnI (ng/mL)^a^	1.26 (1.07–1.48)	0.006	1.218 (1.011–1.468)	0.038	1.239 (1.024–1.499)	0.028
*Variables at the initiation of CRRT*						
APACHE II	1.09 (1.04–1.15)	0.000	1.087 (0.989–1.195)	0.083	1.086 (0.985–1.199)	0.099
SOFA	1.27 (1.14–1.42)	0.000	1.042 (0.767–1.415)	0.794	1.043 (0.762–1.429)	0.792
Time from admission to CRRT initiation (h)	1.01 (1.00–1.02)	0.038	0.998 (0.988–1.007)	0.621	0.99 (0.977–1.002)	0.111
Late CRRT^a^	2.31 (1.13–4.72)	0.002	3.082 (1.072–8.859)	0.037	–	–
Time from admission to the peak CK occurrence(h)^a^	1.016 (1.004–1.029)	0.010	–	–	1.026 (1.004–1.049)	0.023
Sodium (mmol/L)	1.04 (1.001–1.07)	0.045	1.023 (0.98–1.069)	0.294	1.023 (0.979–1.069)	0.317
Albumin (g/L)	0.90 (0.85–0.99)	0.000	0.954 (0.813–1.119)	0.563	0.949 (0.805–1.118)	0.531
Globulin (g/L)	1.09 (1.00–1.17)	0.036	0.981 (0.779–1.234)	0.868	0.969 (0.762–1.233)	0.798

APACHE II: Acute Physiology and Chronic Health Evaluation II; SOFA: Sequential Organ Failure Assessment; CRRT: continue renal replacement therapy; CK: creatine kinase; cTnI: cardiac troponin I; late CRRT: CRRT initiation before 4.8 h after the peak CK occurrence.

Normal range of CK: 40–200 IU/L; normal range of cTnI: 0–0.03 ng/mL; normal range of albumin: 40–55 g/L.

^a^ Both in univariate logistic regression and multivariate analyses, survivors vs. mortality, *p* < 0.05.

The multivariate logistic regression analysis in model one showed that the need for mechanical ventilation support (OR = 4.632, 95% CI 1.292–16.61, *p* = 0.019), elevated serum cTnI at admission (per 1 ng/mL, OR = 1.218, 95% CI 1.011–1.468, *p* = 0.038), and late CRRT (OR 3.082, 95% CI 1.072–8.859, *p* = 0.037) were independent risk factors for 90-day mortality (Table 3). Moreover, the multivariate logistic regression analysis in model two showed that age (per 1 year, OR 1.042, 95% CI 1.003–1.081, *p* = 0.032) and time from CRRT initiation to the peak CK occurrence (per 1 h, OR 1.026, 95% CI 1.004–1.049, *p* = 0.023) were independent risk factors for 90-day mortality.

**Figure 2.**
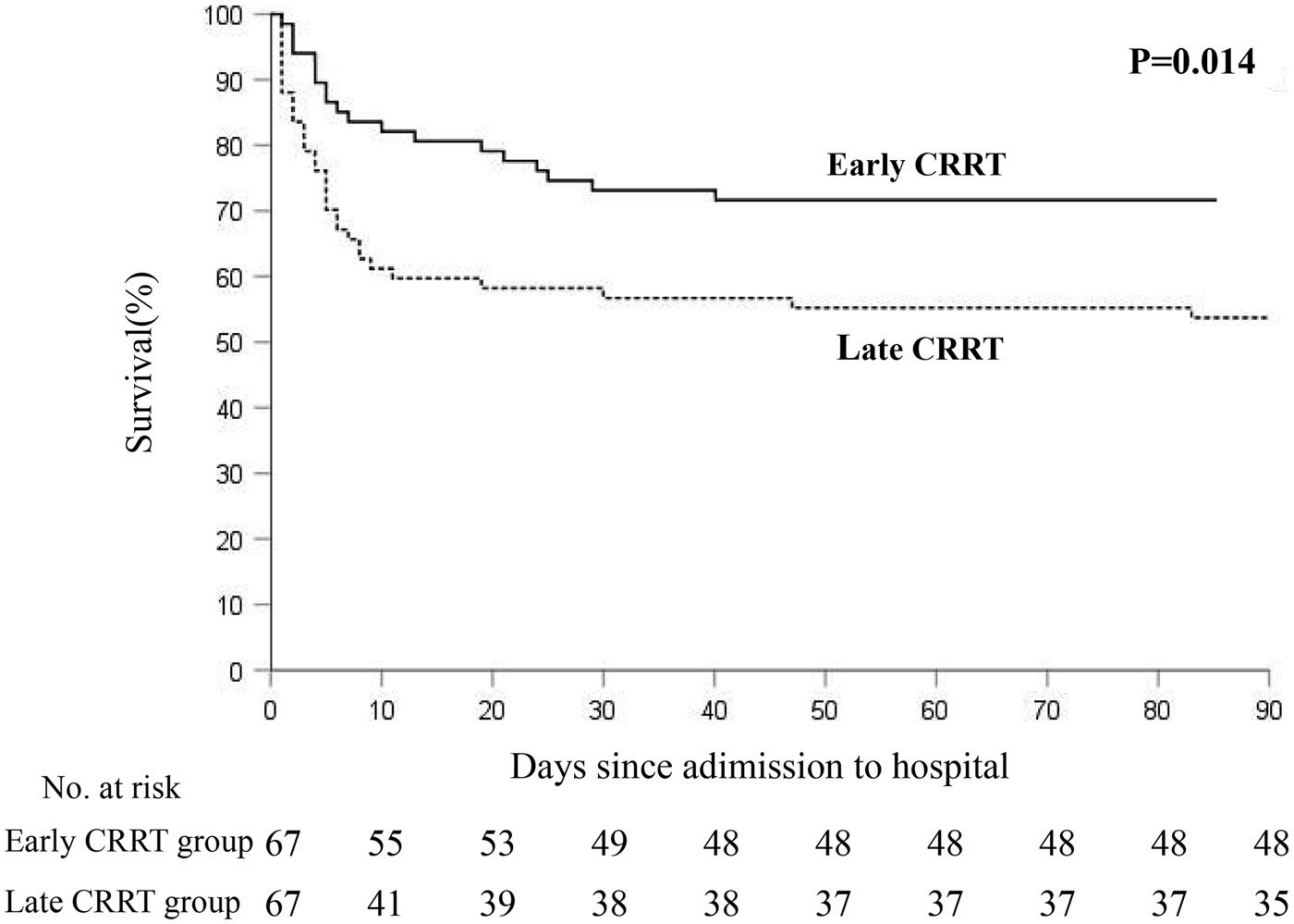
Early CRRT: CRRT initiation before 4.8 h after the peak CK occurrence; late CRRT: CRRT initiation beyond 4.8 h after the peak CK occurrence.

The median follow-up time was 58.9 (33.8, 71.0) months. During the follow-up time, 40.3% (*n* = 54) of patient deaths were observed. Almost all of the deaths occurred within 90 days after hospital admission. The 90-day mortality rate was 38.06% (*n* = 51), with 19 of 67 (28.4%) patients in the early CRRT group and 32 of 67 (47.8%) patients in the late CRRT group. Kaplan–Meier’s survival curves showed that the late CRRT group had significantly increased mortality risk during the follow-up (log-rank test, *p* = 0.014, Figure 2).

### Early group versus late group

The characteristics of patients in the early and late groups are shown in Table 4. The univariate analysis results demonstrated that patients in both groups were similar in age, the use of mechanical ventilation, time from admission to CRRT initiation, mean CK peak, and other known risk factors for mortality, which ensures comparability between the two groups of patients. While patients treated early had lower in-hospital mortality, significantly lower 90-day mortality (*p* = 0.021), and a longer length of stay.

**Table 4.** Comparison of characteristics between patients in early CRRT group and late CRRT group.

Characteristics	Early CRRT (*n* = 67)	Late CRRT (*n* = 67)	*p* Value
*Variables at admission to hospital*			
Age	49.1 ± 17.7	48.8 ± 19.6	0.915
Use of mechanical ventilation (*n*, %)	30 (44.8%)	31 (46.3%)	0.862
Use of vasoactive drugs (*n*, %)	23 (34.3%)	27 (40.3%)	0.475
*Comorbidity, n (%)*			
Diabetes (*n*, %)	11 (16.4%)	9 (13.4%)	0.628
Hypertension (*n*, %)	29 (29.9%)	13 (19.4%)	0.160
Cardiovascular disease (*n*, %)	9 (13.4%)	6 (9.0%)	0.411
APACHE II	16.7 ± 7.8	15.3 ± 8.2	0.313
SOFA	6.9 ± 3.2	7.2 ± 3.6	0.565
*Variables at the initiation of CRRT*			
APACHE II	17.57 ± 7.84	15.5 ± 8.4	0.133
SOFA	8.45 ± 3.62	8.32 ± 4.2	0.844
Time from admission to CRRT (h)	24.5 (17.5, 42.5)	37 (23, 49.5)	0.121
Peak CK (IU/L)	18,513 (4968, 48,826)	12,899 (5033, 42,542)	0.313
*AKI stage at the initiation of CRRT*			0.183
AKI 0 (*n*, %)	10 (14.9%)	13 (19.4%)	
AKI I (*n*, %)	30 (44.8%)	37 (55.2%)	
AKI II, III (*n*, %)	27 (40.3%)	17 (25.4%)	
*Outcomes*			
Length of hospitalization (days)	13 (7, 20)	7 (4, 16)	0.006
In hospital mortality (*n*, %)	6 (9.0%)	14 (20.9%)	0.052
90-day mortality (*n*, %)	19 (28.4%)	32 (47.8%)	0.021

Early CRRT: CRRT initiation before 4.8 h after the peak CK occurrence; late CRRT: CRRT initiation beyond 4.8 h after the peak CK occurrence; APACHE II: Acute Physiology and Chronic Health Evaluation II; SOFA: Sequential Organ Failure Assessment; CRRT: continue renal replacement therapy; CK: creatine kinase; AKI: acute kidney injury.

Normal range of CK: 40–200 IU/L; normal range of cTnI: 0–0.03 ng/mL; normal range of albumin: 40–55 g/L.

### Subgroup analysis

**Figure 3.**
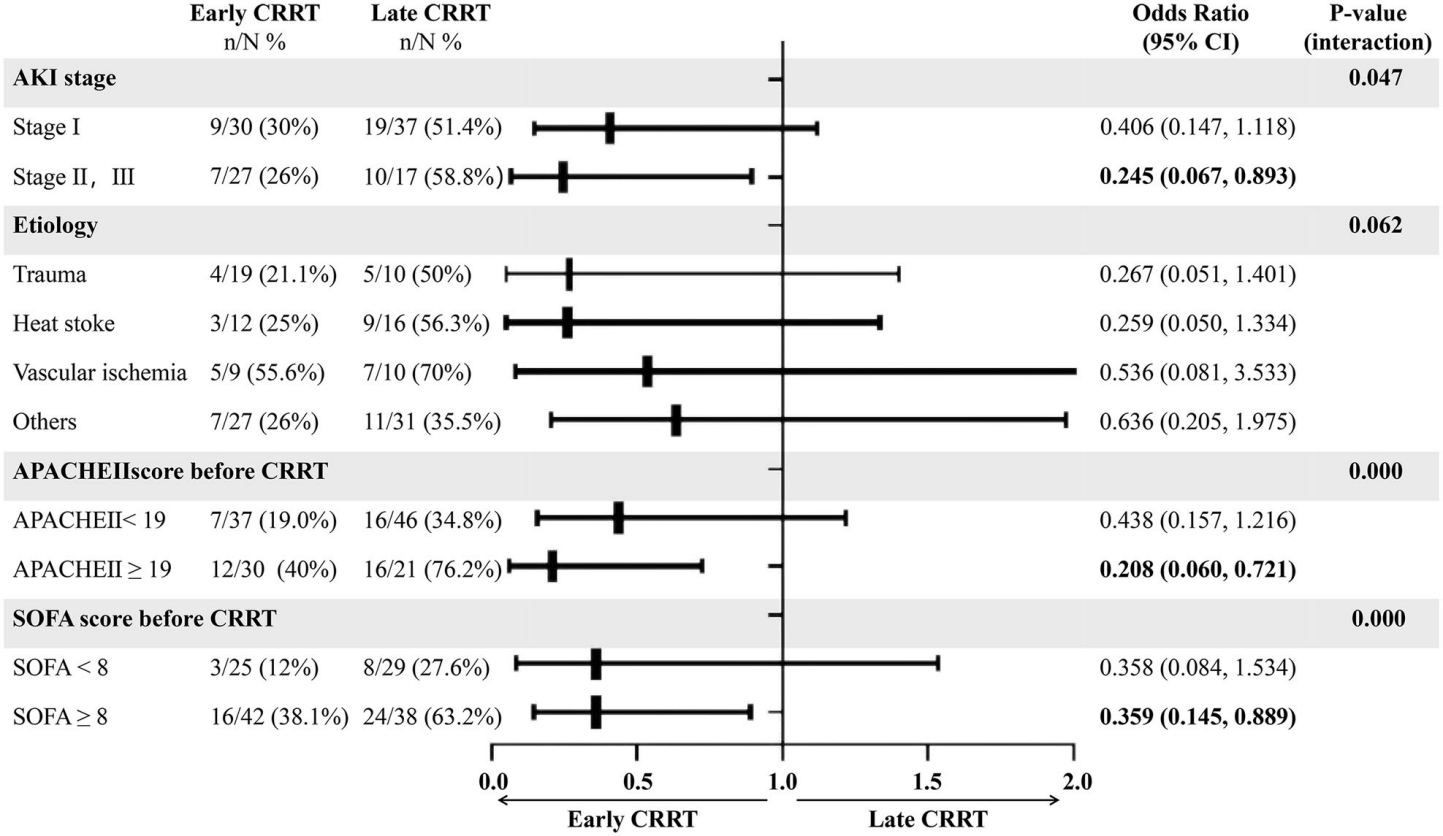
Subgroup analysis. *N*: the number of patients in this subgroup who had early or late CRRT; *n*: the number of patients who died; APACHE II: Acute Physiology and Chronic Health Evaluation II; SOFA: Sequential Organ Failure Assessment; AKI: acute kidney injury; CRRT: continue renal replacement therapy.

Based on the results of the subgroup analysis, we found that the late group was associated with increased mortality in RM patients with severe AKI (stage II or III), higher APACHE II scores (≥19), and higher SOFA scores (≥8) (Figure 3).

## Discussion

To our knowledge, this is the first study to explore the effect of CRRT initiation time on patient mortality and the risk factors for death in RM patients who underwent CRRT. We identified a high short-term mortality rate in this group of patients. The main finding of our study was that time from CRRT initiation to the peak CK occurrence was independently associated with mortality, and relatively earlier CRRT initiation (before 4.8 h after the peak CK occurrence) was associated with a lower risk of 90-day mortality. Moreover, the older age, the requirement for mechanical ventilation, and the elevated serum cTnI concentrations were identified as independent risk factors for 90-day mortality.

RM is a common severe clinical presentation with complicated etiologies. The causes of RM were different among various studies [5,12,27]. In our present cohort, the common causes were trauma, heat stroke, and vascular ischemia. Trauma and vascular ischemia have been widely reported as major causes of RM [28,29]. Compared with previous studies, a higher proportion of heat stroke was found in our cohort. Due to the inclusion of RM patients who received CRRT, the incidence of AKI (82.84%) in our cohort was dramatically higher than that in previous reports. Half of the patients needed aggressive therapies, such as mechanical ventilation (45.52%), vasoactive drugs (37.31%), and blood transfusion (60.3%). Most likely, the disease severity of the included patients was the main contributor to the relatively high 90-day mortality rate (38.1%), which is in the upper range of the previous reports (20.5–40%) [12,29]. Although the cases collected in our study spanned a long period, 126 (94%) of these events occurred after year 2013, and the clinical practice of CRRT in RM patients has not changed significantly over the years [19,21].

CRRT is increasingly used in critically ill patients as a part of organ support. There remain no clear criteria for when to initiate CRRT in clinical practice. The timing of CRRT for AKI has been evaluated by several high-quality RCTs, but the conclusions are controversial. The ELAIN trial, which randomly assigned patients who met KDIGO stage 2 AKI criteria to early RRT (treated within 8 h) or late RRT (treated more than 12 h after the patient reached KDIGO stage 3 AKI), showed that mortality at 90 days, all-cause mortality at 1 year, and rate of renal function unrecovered were significantly lower in the early group [30]. However, three other studies (AKIKI, STARRK, and IDEAL) did not observe increased patient survival in the early CRRT group [31–33]. These results seem to demonstrate that expectant management may be acceptable when RRT is not urgently indicated and when there is uncertainty about whether AKI will recover without RRT [34]. In the recent AKIKI2 trial, the timing of treatment was further delayed in the late RRT group (treatment was initiated after 72 h of oliguria or with a blood urea nitrogen level exceeding 112 mg/dL and the emergence of required indications), and the result showed that a more delayed approach was associated with worse outcomes [35].

Premature CRRT may expose patients to the risk of additional CRRT-related complications. Nevertheless, delayed initiation of CRRT might increase the risk of organ injury by fluid overload, acid–base, and electrolyte disorder [36,37]. Meanwhile, in the absence of alternative treatment, these patients need to use many drugs to control these complications, and the side effects of these drugs are also potential risks that need to be considered [38]. In summary, the appropriate timing of RRT still needs further exploration and individualized customization [38].

Compared to AKI patients with other etiologies, patients with RM-induced AKI are more likely to profit from CRRT by the continuous clearance of serum MB and other toxic substances released from damaged myocytes. To date, no reports have specifically evaluated the impact of the timing of CRRT initiation on the prognosis of RM patients. However, risk factors for patients who may require RRT have been reported.

CK is an important substance released from damaged myocytes [39], which has been widely discussed as a useful indicator in the prognostic evaluation of RM patients for the following reasons. First, CK is the most sensitive biomarker of muscle injury, it does not damage the organism, and can react to the degree of injury. Byerly et al. found that patients with higher serum peak CK had more severe muscle injuries, greater injury severity score, and a higher incidence of bone fractures [40]. Second, CK rises within 12 h after the onset of RM and peaks within 24–72 h, and the longer half-life of CK offers a wider observation time window, and testing CK has the advantage of lower cost and wide availability [39,41]. Third, previous studies have demonstrated a correlation between elevated CK and poor prognosis in RM patients. In a large cohort of 3111 trauma patients, Harrois et al. reported that the peak CK value was significantly higher in patients with risk, injury, and failure-stage kidney disease than in patients without AKI (2800 IU/L vs. 977 IU/L, *p* < 0.001) [42].

CK levels were also reported to be associated with the need for CRRT. In a retrospective study of 2371 RM patients, McMahon et al. developed a prediction model of RRT or mortality using eight clinical variables, which included CK values [12]. In another forecasting model, CK was also included as a factor in assessing the demand for RRT [43]. However, a recent report showed that in patients who underwent CRRT and recovered renal function, higher CK values at CRRT termination were not associated with an increased need for RRT, which indicated that CK is not an effective indicator for CRRT termination. At the end of this paper, the authors suggested prospective clinical trials to investigate and confirm the optimal CK range that could be used as a guideline [44]. In summary, the predictive role in the RRT requirement of CK needs to be further explored.

In the present study, we observed that the time from CRRT initiation to the peak CK occurrence was an independent risk factor for mortality. The later the initiation of CRRT after the appearance of peak CK, the higher the risk of patient mortality. We classified participants into early and late groups based on the median time from CRRT initiation to the peak CK and found lower mortality in the early group. Moreover, there were no significant differences in APACHE II scores, SOFA scores, or laboratory parameters between the two groups at admission and before CRRT, which indicates that the two groups of patients were similar.

Peak CK appears 24–72 h after muscle injury, and persistently elevated CK indicates ongoing muscle injury or the development of renal failure [45], CK peak time is theoretically related to the muscle injury time and severity. Initiating CRRT before or soon after peaking could be considered as an earlier strategy, compared to CRRT initiation late after peak CK occurrence. Additionally, the employment of CRRT does not directly change the natural progression of CK value, due to CK is a large molecule with a molecular weight of 87 kDa and cannot be removed by CRRT with conventional hemodialysis filters [46,47]. Therefore, the dynamic changes in CK values that we observed are objectively related to the progression of the disease rather than to the dose and timing of CRRT, which provides us with an objective judgment of the timing of CRRT based on the peak CK or dynamic changes in CK in RM patients.

Early initiation of CRRT may provide potential benefits to patients through the following mechanisms. First, the low efficiency of MB clearance leads to high circulating levels of the molecule, resulting in renal injury and delayed recovery of renal function [19]. Earlier CRRT contributes to lower circulating levels of MB, reduces MB deposition in the renal tubules, and attenuates MB-induced lipid peroxidation and mitochondrial damage [16]. Moreover, in the earlier phase of RM, the systemic inflammatory response is activated [48]. The increase in CK and MB is accompanied by a gradual rise in various inflammatory cytokines, such as tumor necrosis factor-α (TNF-α), interleukin-1β (IL-1β), and interleukin-6 (IL-6) [49]. It has been reported that CRRT can reduce serum inflammatory cytokines and facilitate immunomodulation [50,51]. Therefore, earlier CRRT most likely could benefit RM patients by clearing MB and alleviating the inflammatory response.

In conclusion, our findings provide theoretical support that early CRRT may improve the prognosis of RM patients. However, it is also important to prevent and control the potential risks of CRRT, such as anticoagulation related complications. Appropriate anticoagulation, careful monitoring, and standardized procedure are required to reduce the CRRT related complications. Future studies are needed to explore more appropriate timing of treatment for patients with RM by balancing the benefits of early CRRT and the risks of early CRRT.

The requirement for mechanical ventilation, serum cTnI concentration, and older age were identified as independent risk factors for 90-day mortality in our present study. Changes in the structure and function of the kidney of the elderly make them vulnerable to toxins released from muscle destruction, and they often have poor physical condition and reduced organ compensatory ability, which increase the risk of mortality [52]. Previous studies have shown that the need for mechanical ventilation in patients who undergo RRT is an independent risk factor for mortality [53]. In our cohort, the requirement for mechanical ventilation, which indicated a severe physical condition and additional organ involvement, was understandable to be identified as one of the independent risk factors for 90-day mortality.

Previous studies have found elevated cTnI in patients with RM [54,55], which indicates cardiac involvement and is associated with poor prognosis [56]. In a cohort study of 178 RM patients, high accuracy of hs-cTnI was reported for the prediction of in-hospital death or prolonged hospital stay (AUC 0.74; 95% CI 0.68–0.80) [57]. The causes of RM, including sepsis, stroke, medications, and trauma, may lead to microinjury of the myocardium, which is the major cause of elevated cTnI [58]. Moreover, the massive release of cytokines, oxygen free radicals, and activation of the systemic inflammatory response in RM patients could result in myocardial cell damage [55]. In RM patients treated with CRRT, volume overload and ischemic-hypoxic injury to the myocardium could also be considered potential causes of elevated cTnI. These relationships most likely explain the identification of cTnI as one of the independent risk factors for 90-day mortality in our present study. The routine monitoring of cardiac function would be helpful for RM patients with or without CRRT.

Our present study has several limitations. First, this is a single-center study, and the disease spectrum might be different from that of other centers. A multicenter study should be performed to validate our findings. Second, although we reported a large cohort of RM patients treated with CRRT, the overall sample size was relatively small. Therefore, a type II error needs to be considered when interpreting our results. Third, although the time of CK peak is a retrospective parameter, our results first suggested that early CRRT most likely could benefit patients with RM and warranted further exploration of CRRT timing based on the dynamic change of CK and patient severity.

## Conclusions

Earlier CRRT is associated with reduced patient mortality. Patients with RM may benefit from dynamic monitoring of changes in CK values and timely initiation of CRRT. Further studies are warranted to identify the CRRT timing for RM patients.

## Data Availability

The datasets used and/or analyzed during the current study are available from the corresponding author on reasonable request.
